# Timing of debridement, antibiotics, and implant retention (DAIR) for early post-surgical hip and knee prosthetic joint infection (PJI) does not affect 1-year re-revision rates: data from the Dutch Arthroplasty Register

**DOI:** 10.5194/jbji-6-329-2021

**Published:** 2021-09-03

**Authors:** Barry van der Ende, Jakob van Oldenrijk, Max Reijman, Peter D. Croughs, Liza N. van Steenbergen, Jan A. N. Verhaar, P. Koen Bos

**Affiliations:** 1 Department of Orthopaedic Surgery and Sport Medicine, Erasmus University Medical Center, Rotterdam, 3000 CA, the Netherlands; 2 Department of Microbiology, Erasmus University Medical Center, Rotterdam, 3000 CA, the Netherlands; 3 Dutch Arthroplasty Register (Landelijke Registratie Orthopedische Implantaten), 's Hertogenbosch, 5232 AD, the Netherlands

## Abstract

Debridement, antibiotics, and implant retention (DAIR) is a procedure to treat a periprosthetic joint infection (PJI) after total hip arthroplasty (THA) or total knee arthroplasty (TKA). The timing between the primary procedure and the DAIR is likely a determinant for its successful outcome. However, the optimal timing of a DAIR and the chance of success still remain unclear. We aimed to assess the risk of re-revision within 1 year after a DAIR procedure and to evaluate the timing of the DAIR in primary THA and TKA. We used data from the Dutch Arthroplasty Register (LROI) and selected all primary THA and TKA in the period 2007–2016 which underwent a DAIR within 12 weeks after primary procedure. A DAIR was defined as a revision for infection in which only modular parts were exchanged. A DAIR was defined as successful if not followed by a re-revision within 1 year after DAIR; 207 DAIRs were performed <4 weeks after THA, of which 16 (8 %) received a complete revision within 1 year. DAIR procedures performed between 4 and 12 weeks (n=98) had a failure rate of 9 % (n=9). After TKA 126 DAIRs were performed in less than 4 weeks, of which 11 (9 %) received a complete revision within 1 year; 83 DAIRs were performed between 4 and 12 weeks, of which 14 (17 %) were revised.

There was no significant difference in 1-year re-revision rate after a DAIR procedure by timing of the DAIR procedure for total hip and knee arthroplasty based on Dutch registry data.

## 1 Introduction

Periprosthetic joint infection (PJI) following hip and knee replacement surgery is a catastrophic complication with an incidence of 1 %–2 % (Zimmerli et al., 2004; Kuiper et al., 2014; Sendi et al., 2017). With an increasing annual number of total joint replacements, the absolute annual incidence of PJI is increasing accordingly (Zimmerli et al., 2004; Kuiper et al., 2014).

The leading treatment strategy for an acute postoperative PJI is debridement, antibiotics, and implant retention or “DAIR procedure” (Tsukayama et al., 1996; Zimmerli et al., 2004; Toms et al., 2006; Kuiper et al., 2014; Sendi et al., 2017) according to most guidelines. Exchange of modular components as part of a DAIR procedure is recommended in total hip arthroplasty (THA) as well as total knee arthroplasty (TKA) (Qasim et al., 2017; Tsang et al., 2017; Parvizi et al., 2019).

A few retrospective series have described an implant retention rate of 60 %–80 % 1 year after treatment of acute PJI (Kuiper et al., 2013, 2014; Anagnostakos and Schmitt, 2014; de Vries et al., 2016; Qasim et al., 2017; Tsang et al., 2017). The group of Trampuz suggested that DAIR is the preferred treatment of an acute PJI only within the first 4 weeks after joint replacement, according to biofilm theory (Izakovicova et al., 2019), whilst according to de Vries et al. (2016), risk of revision surgery is increased in case DAIR is performed later than 3 months after index surgery for both THA and TKA. However, in a retrospective study of 89 PJIs, Geurts et al. (2013) showed that implants could be preserved in 50 % of patients when the DAIR procedure was performed 8 weeks after joint replacement.

Clearly, a DAIR procedure for a PJI is effective shortly after surgery. Whether it is also a viable option in patients with a delayed presentation is unknown. Could implants be preserved with a DAIR even up to 3 months? To find a more pronounced relation between outcome of treatment and the period between primary procedure and DAIR, a large number of patients is needed which cannot be reached in a single centre. Therefore, data from the Dutch Arthroplasty Register (LROI) were studied.

In this study we aimed to determine the DAIR failure rate within 1 year in patients who had a DAIR procedure within 4 weeks after a primary procedure compared to a DAIR procedure 4–12 weeks for acutely postoperative infected primary implants. Furthermore, independent risk factors for re-revision after a DAIR procedure were examined.

## 2 Methods

The LROI is a nationwide population-based register that has included information on arthroplasties in the Netherlands since 2007. The LROI was initiated by the Dutch Orthopaedic Association and the database has 100 % hospital coverage for hip and knee arthroplasty, with a 99 % completeness for both primary total hip and primary total knee arthroplasty (LROI, 2019). The LROI database contains information on patient, procedure, and arthroplasty characteristics based on product numbers registered on the day of surgery (van Steenbergen et al., 2015). Body mass index (BMI) and smoking status were added to the LROI in 2014. The vital status of all patients is obtained actively on a regular basis from Vektis, the national insurance database on healthcare in the Netherlands, which records all deaths of Dutch citizens (http://www.Vektis.nl, last access: 29 October 2018). The LROI uses the opt-out system to require informed consent of patients.

For the present study we included all primary THAs and TKAs for osteoarthritis in the period 2007–2016, which were partially revised within 12 weeks after the primary implantation for infection or the suspicion thereof. A partial revision was defined here as a revision procedure where at least the femoral head (with or without the acetabular liner) in THA or the insert in TKA were exchanged. With this definition based on the LROI we presumed to include all patients undergoing a DAIR with exchange of modular components for acute PJI registered in the LROI.

We divided both hip and knee DAIR procedures into two groups according to time between index surgery and DAIR procedure. Group A underwent a DAIR procedure within 4 weeks, Group B between 4 and 12 weeks after the index procedure.

We defined failure of a DAIR procedure as a complete re-revision within 1 year after the DAIR procedure. A complete revision was defined as the replacement or removal of all components for any reason, as definitive treatment or as part of either a single stage or multiple-stage revision.

For secondary analysis we used any type of revision within 1 year, including repeat-DAIR procedures.

### 2.1 Statistical analysis

Chi-squared tests were performed to test any differences in failure of DAIR procedures between the two time period groups, stratified by joint. Multivariable logistic regression analysis was performed to examine independent risk factors for re-revision after a DAIR procedure. The dependent variable was failure of a DAIR procedure within 1 year, and independent variables were age, gender, American Society of Anesthesiologists (ASA) classification, and type of fixation (SPSS 22.0; IBM Corp., Armonk, NY, USA).

P values below 0.05 were considered statistically significant. For the 95 % confidence intervals (CIs), we assumed that the number of observed cases followed a Poisson distribution.

### 2.2 Potential conflicts of interest and statement of ethics

Our study was approved by the scientific advisory board of the LROI. The dataset was processed according to LROI regulations on research using registry data. Ethical approval was not requested, since these data were collected as part of the usual (evaluation of) care process. No funding was received and no conflicts of interests were declared. All the authors contributed equally.

**Figure 1:**
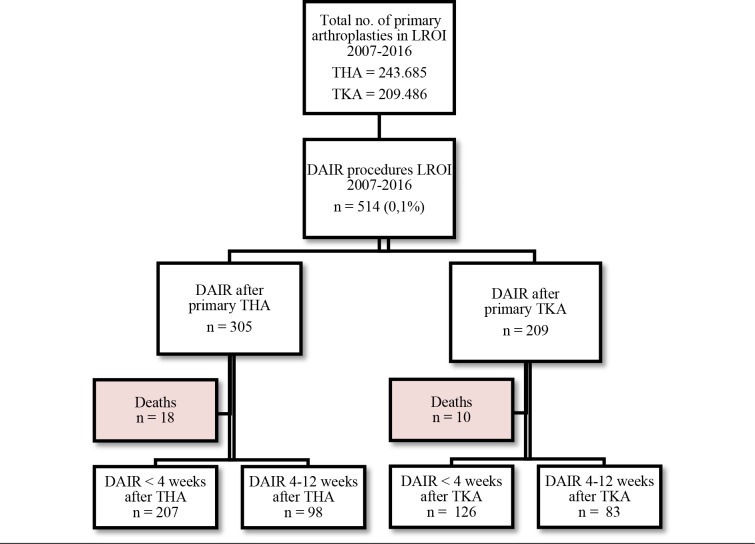
Patient flow.

## 3 Results

Between 1 January 2007 and 31 December 2016, 514 patients fulfilled the inclusion criteria for a DAIR procedure: 305 after a primary THA and 209 after a primary TKA (Fig. 1). In the THA group, 18 (5.9 %) deaths were registered, of which 4 (1.3 %) occurred within 1 year after a DAIR procedure. In the TKA group, 10 (4.8 %) deaths were registered during our study period, of which 5 (2.4 %) were within 1 year after a DAIR procedure. None of these patients had undergone a re-revision prior to death. They were excluded from further analysis.

Patient characteristics for THA and TKA with a subsequent DAIR procedure were comparable, with an average age of 69 years (Table 1).

**Table 1:** Patient characteristics of the study population during the primary procedure (n=514).

	THA	TKA
	(n=305)	(n=209)
Age (years) (mean, SD)	69 (10)	69 (10)
Gender (n, %) female	163 (53)	88 (42)
ASA classification (n, %)		
ASA I	42 (14)	34 (16)
ASA II	199 (65)	126 (60)
ASA III–IV	62 (20)	47 (23)
BMI category (n, %)		
Underweight (<18.5 kg/m${}^{2}$)	1 (0.3)	–
Normal (18.5–25)	42 (14)	27 (13)
Overweight (25–30)	111 (36)	67 (32)
Obesity (30–40)	110 (36)	60 (29)
Morbid obesity (>40)	12 (4)	12 (6)
Smoking (n, %)		
Yes	32 (11)	18 (9)
No	227 (74)	130 (62)
Type of fixation (n, %)		
Cemented	100 (33)	201 (96)
Uncemented	155 (51)	5 (2)
Hybrid	50 (16)	3 (1)

BMI: body mass index. ASA classification: American Society of Anesthesiologists physical status classification. NB: numbers do not add up to the total due to missing data, BMI, and smoking registered since 2014.

**Figure 2:**
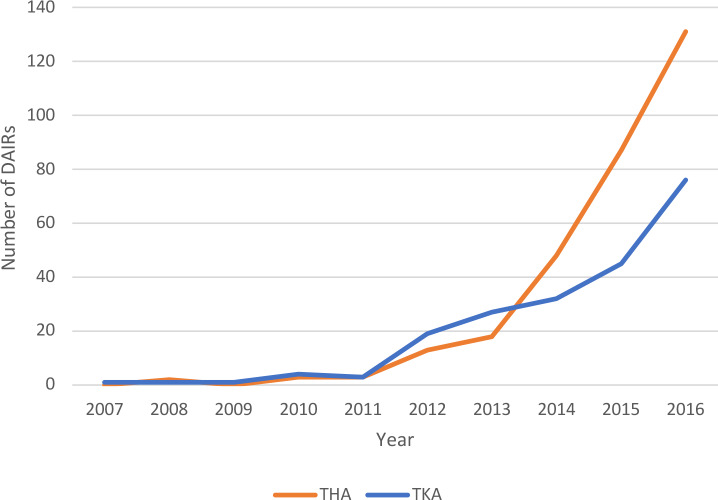
Trend of DAIRs (with modular component exchange) registered per year in the LROI (x axis: year in which procedure was registered, y axis: number of DAIRs registered).

The number of registered DAIR procedures with modular component exchange has gradually increased since 2007. Up to 2011, less than 10 DAIR procedures per year were registered after THA and TKA each nationwide. These numbers increased to 48 (16 %) in 2014 up to 131 (43 %) in 2016 for THA. For TKA the number of registered DAIRs increased to 32 (15 %) in 2014 and up to 76 (36 %) TKAs in 2016 (Fig. 2).

The large majority (91 % in THA and 82 % in TKA) of re-revisions within 1 year were performed for infection. Dislocation, periprosthetic fracture, and loosening of the acetabular component were alternative reasons for revision in 9 % of cases in THA. In TKA other reasons for re-revision were loosening, instability or malalignment, and arthrofibrosis.

### 3.1 Total hip arthroplasties

There were 207 THA patients that underwent a DAIR procedure within 4 weeks after index surgery (Group A) and 98 patients between 4 and 12 weeks (Group B) (Table 2).

**Table 2:** Number of re-revisions after DAIR procedures with odd ratios for different timings of DAIR after primary THA.

	Complete revision	OR (CI)	Re-revision <1 year	OR (CI)
	<1 year		(incl. repeat-DAIR)	
DAIR <4 weeks (n=207)	16 (8 %)	Ref${}^{*}$	41 (20 %)	Ref
DAIR 4–12 weeks (n=98)	9 (9 %)	1.2 (0.5–2.9)	17 (17 %)	0.9 (0.5–1.6)
Total	25 (8 %)		58 (19 %)	
ASA class				
ASA I (n=42)	5 (12 %)	0.8 (0.2–3.8)	6 (14 %)	0.3 (0.1–1.9)
ASA II (n=199)	23 (12 %)	Ref	43 (22 %)	Ref
ASA III–IV (n=62)	4 (6 %)	0.3 (0.1–1.8)	8 (13 %)	0.4 (0.1–2.4)
Gender				
Female (n=163)	11 (7 %)	Ref	18 (11 %)	Ref
Male (n=140)	22 (16 %)	2.6 (1.2–5.5)	39 (28 %)	3.1 (1.7–5.7)
Age (years)				
<70 (n=164)	20 (12 %)	Ref	37 (23 %)	Ref
>70 (n=141)	13 (9 %)	0.7 (0.4–1.5)	21 (15 %)	1.5 (0.8–2.8)
Type of fixation				
Cemented (n=100)	9 (9 %)	Ref	14 (14 %)	Ref
Uncemented (n=155)	19 (12 %)	1.4 (0.6–3.3)	37 (24 %)	1.8 (0.9–3.5)
Hybrid (n=50)	5 (10 %)	1.0 (0.3–3.2)	7 (14 %)	0.9 (0.3–2.4)

${}^{*}$ Ref: OR reference group; numbers do not add up to the total due to missing data.

**Figure 3:**
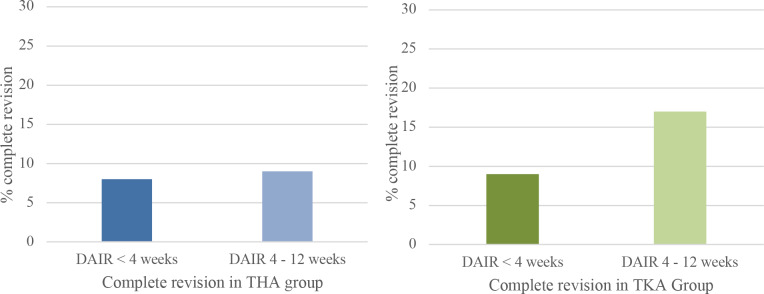
Percentage of complete revision within 1 year when DAIR is performed for acute postoperative PJI <4 weeks after primary surgery or between 4 and 12 weeks. Blue graph for THA and green for TKA.

**Figure 4:**
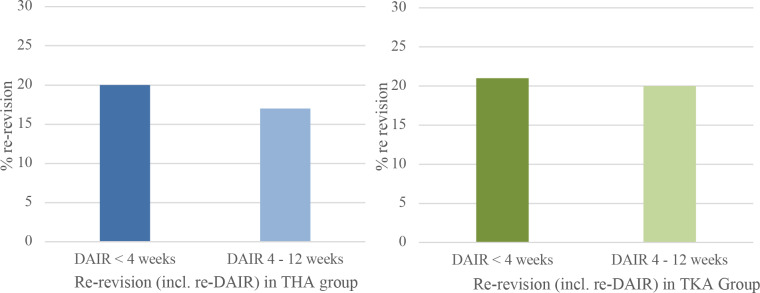
Percentage of re-revision including repeat-DAIR procedure within 1 year when DAIR is performed for acute postoperative PJI <4 weeks after primary surgery or between 4 and 12 weeks. Blue graph for THA and green for TKA.

A complete revision (exclusive repeat-DAIR procedure) rate of 8 % (n=16) was found in Group A and 9 % (n=9) in Group B (Fig. 3). When repeat-DAIR procedures were included, in Group A 20 % (n=41) received a re-revision within 1 year, in Group B 17 % (n=17) (Fig. 4).

Male gender showed an increased risk of a complete re-revision, with an OR of 2.6 (CI 1.2–5.5) and an odds ratio of 3.1 (CI 1.7–5.7) when repeat-DAIR procedures are included. Neither ASA class, age, nor type of fixation were significantly associated with the primary endpoint (Table 2).

### 3.2 Total knee arthroplasties

After TKA, 126 DAIR procedures were performed within 4 weeks (Group A) and 83 DAIRs were performed after 4–12 weeks (Group B) (Table 3).

**Table 3:** Number of re-revisions after DAIR procedures with odd ratios for different timings of DAIR after primary TKA.

	Complete revision	OR (CI)	Re-revision <1 year	OR (CI)
	<1 year		(incl. repeat-DAIR)	
DAIR <4 weeks (n=126)	11 (9 %)	Ref${}^{*}$	27 (21 %)	Ref
DAIR 4–12 weeks (n=83)	14 (17 %)	2.1 (0.9–4.9)	17 (20 %)	0.9 (0.5–1.9)
Total	25 (12 %)		44 (21 %)	
ASA class				
ASA I (n=34)	1 (3 %)	0.2 (0.02–1.2)	6 (18 %)	0.7 (0.3–1.9)
ASA II (n=126)	19 (15 %)	Ref	28 (22 %)	Ref
ASA III–IV (n=47)	9 (19 %)	1.4 (0.6–3.3)	9 (19 %)	0.8 (0.4–1.9)
Gender				
Female (n=88)	11 (13 %)	Ref	14 (16 %)	Ref
Male (n=121)	19 (16 %)	1.2 (0.6–2.8)	30 (25 %)	1.7 (0.8–3.4)
Age				
<70 (n=116)	17 (15 %)	Ref	25 (22 %)	Ref
>70 (n=93)	13 (14 %)	1.1 (0.5–2.4)	19 (20 %)	1.1 (0.5–2.1)
Type of fixation				
Cemented (n=201)	19 (9 %)	Ref	42 (21 %)	Ref
Uncemented (n=5)	0 (0 %)	–	1 (20 %)	0.9 (0.1–8.1)
Hybrid (n=3)	0 (0 %)	–	1 (33 %)	1.8 (0.2–20.0)

${}^{*}$ Ref: OR reference group; numbers do not add up to the total due to missing data.

In Group A the number of patients undergoing a complete revision was 9 % (n=11) compared to 17 % (n=14) in Group B, which did not show a statistically significant difference (Fig. 3). In Group A 27 patients (21 %) received a re-revision (including repeat-DAIRs) within 1 year, in Group B 17 (20 %) (Fig. 4).

No independent risk factor (ASA score, gender, age, or type of fixation) could be identified, resulting in an increased risk of re-revision (Table 3).

We pooled data for both THA and TKA DAIR procedures, showing an 8 % complete revision rate when DAIR was within 4 weeks and 13 % when DAIR was within 4–12 weeks. Including repeat-DAIR procedures, the re-revision rate for the group <4 weeks was 20 %, and for the group 4–12 weeks it was 19 %. There was no significant difference between groups in the pooled data.

## 4 Discussion

In our observational LROI data analysis, we did not find an increase in DAIR failure rate when a DAIR is performed between 4 weeks and up to 12 weeks after primary surgery for early hip or knee arthroplasty PJI.

The Dutch national implant registry data do not support the theory that a DAIR procedure can only treat a PJI when performed within 4 weeks after primary surgery. We could not show that the success of a DAIR procedure decreases when a larger interval exists between DAIR and primary procedure (Izakovicova et al., 2019). Our findings were supported by the recently published retrospective study by Löwik et al. (2020) including 769 PJIs.

Based on the discussion in the literature on the definition of acute implant infections (Tsukayama et al., 1996; Zimmerli et al., 2004; Toms et al., 2006), ranging from up to 3–4 weeks to 3 months, we decided to use a study period ranging from 0 to 12 weeks. We excluded acute hematogenous infections since these patients cannot be identified due to the limited detailed information in registries regarding infections. Infection was the reason for re-revision after a DAIR procedure in 91 % of the cases for hip replacement and in 82 % for knee replacement. In the total hip population there were two cases with acetabular loosening as the reason for a re-revision, although low-grade infection could be an alternative explanation for this early loosening. The same can be said about the cases with loosening of a component or arthrofibrosis in the total knee group. We considered all complete revisions for any reason to be an implant failure.

Because of deaths, 5 %–6 % of patients were excluded from analysis; cause of death is unknown in the LROI. To prove there is no exclusion bias influencing our results, we performed a sensitivity analysis with a worst-case scenario, with every death scoring as a complete revision. This analysis shows an OR of 1.3 (CI 0.7–2.5) when DAIR is performed >4 weeks after THA and an OR 1.8 (CI 0.9–3.6) >4 weeks after TKA, which corresponds to our findings.

We performed this study to investigate the success or failure rate of a DAIR procedure after primary knee and hip replacement in association with timing. The data were selected with a number of assumptions. We trust the considerations by the treating orthopaedic surgeon to perform a DAIR procedure based on a grounded suspicion of a PJI and the assumption of correct registration of procedures. By selecting DAIR procedures from the LROI, we selected only DAIR procedures in which an exchange of modular components was performed. In total knee replacements this means an exchange of the insert, and in total hip replacements this means an exchange of the femoral head and/or acetabular liner. The results presented in this study are only applicable for DAIR procedures with modular component exchange.

The ideal approach in our retrospective study would be to include the investigated variable (time to DAIR) in a multivariate model with other co-variates with a known influence on revision rate (like for instance significant co-morbidity such as rheumatoid arthritis, chronic renal impairment, immunosuppressant therapy, or antibiotics used). However, the registry only includes information regarding gender, BMI, and age, which is a limitation.

We observed a trend of increased component exchange during DAIR procedures. A nationwide Dutch survey showed that 82 % out of 99 institutions exchanged the insert during a DAIR procedure for a TKA infection (Veltman et al., 2018). In THA, 66 out of 99 institutions exchanged all modular parts during a DAIR procedure, 8 institutions routinely exchanged the femoral head only, and 3 exchanged modular parts routinely during the second DAIR procedure (Veltman et al., 2018). As a consequence, the authors showed that 63 % of institutions registered DAIR procedures in the LROI database (Veltman et al., 2018). Some surgeons may choose not to change the modular components, either to prevent additional damage to the component or based on insufficient evidence supporting the exchange of modular components during PJI treatment.

The increasing number of DAIRs being registered over the years in the Dutch registry is most probably explained by the increased awareness of the importance of exchanging the modular components in the literature and (inter)national guidelines. Modular component exchange is shown to have a positive effect on the success rate of DAIR procedures (Argenson et al., 2019; Lora-Tamayo et al., 2013).

The percentage of re-revisions after DAIR procedures is for both total hip and knee arthroplasty around 20 % in our analysis. This number is in accordance with general results after DAIR procedures in the literature, with a success rate of 60 %–80 % (Tsukayama et al., 1996; Zimmerli et al., 2004; Toms et al., 2006; Kuiper et al., 2014; Sendi et al., 2017), and does not differ between groups.

We showed no clear increased re-revision rate after DAIR for all patient characteristics except for male gender after THA. The observed male gender effect in our analysis may be explained by the general increased risk of PJI development in men, as described in the systematic literature review in 2016 by Kunutsor et al. (2016). Perhaps this also explains an increased risk of PJI in post-DAIR patients. Using a cemented or cementless prosthesis does not seem to influence the risk of DAIR failure. We did not make a distinction between antibiotic-loaded or antibiotic-unloaded cement, because the majority (>94 %) of cemented prostheses registered in the LROI were implanted using gentamicin-loaded cement for both THA and TKA (LROI, 2019).

Results in our study can be influenced by unknown confounders such as unknown patient characteristics and the difference in causative microbiological agent and antibiotic regimes following DAIR in different institutions. We assume that the clinical decision to perform a DAIR procedure has been made according to known guidelines and a well-substantiated diagnosis of a PJI given. Some treatment protocols may use a standard second DAIR procedure; this might have influenced our results.

To conclude, based on Dutch Arthroplasty Register data, regarding acutely infected total hip or knee arthroplasties, we were not able to show a difference in re-revision rates for DAIR procedures performed within 4 weeks or between 4 and 12 weeks with 1-year follow-up. In delayed presentations of an acute postoperative PJI, a DAIR procedure may be a viable treatment to preserve the implant at least up to 3 months.

## Data Availability

Data are owned by the Dutch Arthroplasty Register (LROI) and cannot be provided without permission by the scientific board.
